# Identification of pathogenicity determinants in ToLCNDV and their RNAi-based knockdown for disease management in *Nicotiana benthamiana* and tomato plants

**DOI:** 10.3389/fmicb.2024.1481523

**Published:** 2024-11-27

**Authors:** Mehulee Sarkar, Dipinte Gupta, Oinam Washington Singh, Samrat Paul, Ravinder Kumar, Bikash Mandal, Anirban Roy

**Affiliations:** Advanced Centre for Plant Virology, Division of Plant Pathology, ICAR-Indian Agricultural Research Institute, New Delhi, India

**Keywords:** begomovirus, dsRNA, hairpin RNAi, pathogenicity determinant, tomato leaf curl New Delhi virus, virus management

## Abstract

*Begomovirus solanumdelhiense* (tomato leaf curl New Delhi virus, ToLCNDV), is member of the genus *Begomovirus*, family *Geminiviridae*, is a prolific bipartite whitefly transmitted begomovirus in the Indian sub-continent has a wide host range, including solanaceous, cucurbitaceous and other plants. Recently, dsRNA-mediated non-transgenic approaches have been promising in managing plant viruses. Such an approach could be effective if the pathogenicity determinants of a virus are targeted. In the case of ToLCNDV, viral pathogenicity has been demonstrated with coat protein (AV1), pre-coat protein (AV2), transcription activator protein (AC2) and nuclear shuttle protein (NSP). In the present study, we investigated the involvement of the three RNA silencing suppressor proteins (AV2, AC2, AC4) encoded by ToLCNDV in pathogenicity determinants through transient overexpression and hairpin RNAi-based knockdown assays in *Nicotiana benthamiana* plants. Further, we showed that the transcripts of AV2, AC2, and AC4 genes can systemically move and express their proteins. Hairpin RNAi constructs targeting each pathogenicity determinant could effectively reduce symptom development and virus titer upon inoculation of ToLCNDV in *N. benthamiana* plants. Exogenous application of dsRNA individually (dsAV2/dsAC2/dsAC4) or together (cocktail dsRNA: dsCk) against the pathogenicity determinants showed a significant reduction of viral load and reduced severity of disease in plants treated with dsCk followed by dsAC4. The present report reconfirms that the RNA silencing suppressor proteins encoded by DNA-A genomic component of ToLCNDV, can also act as pathogenicity determinants. Further, we demonstrated for the first time that exogenous application of dsRNA targeting those pathogenicity determinants reduces ToLCNDV load and limits symptom development in tomato plants.

## Introduction

Begomoviruses (family *Geminiviridae*) are circular single-stranded DNA viruses, with genome sizes ranging from 2.7 kilobases (monopartite) to 5.4 kilobases (bipartite). They are transmitted by whiteflies (*Bemisia tabaci*), and cause severe damage to economically important crops in tropical and subtropical regions globally (Mansoor et al., 2003). A large number of begomovirus species are also known to occur in India, where they pose a major limiting factor for enhancing the productivity of several vegetable crops (tomato, potato, chilli, okra, pumpkin, cucumber), pulse (mungbean, black gram, French bean, pigeonpea), oilseed (soybean) and fibre crops (cotton, jute, mesta) (Varma et al., 2011). Tomato cultivation in India is particularly affected by a leaf curl disease, which is associated with at least 14 different species of begomoviruses. Among these, tomato leaf curl New Delhi virus (ToLCNDV) (species: *Begomovirus solanumdelhiense*) stands out as the most prevalent and prolific virus, with a wide host range across the country (Varma et al., 2011). Tomato plants infected with ToLCNDV typically exhibit symptoms like vein clearing, leaf puckering, blistering, upward or downward curling, crinkling and reduction in size of leaves (Moriones et al., 2017). Infected plants also showed a reduction in growth and plant height with fewer fruits. It was first reported in tomatoes in India in 1995 (Padidam et al., 1995), and later spread to different cucurbits (Sohrab et al., 2003; Vignesh et al., 2023), potato (Usharani et al., 2004), chilli (Khan et al., 2006), and cotton (Zaidi et al., 2016) in the Indian subcontinent. ToLCNDV is the second most widespread begomovirus, after tomato yellow leaf curl virus (TYLCV) that has no geographical limitations, as it has currently spread to various countries in Asia, North Africa, and Southern Europe (Malathi et al., 2017). ToLCNDV comprises two genomic components, DNA-A and DNA-B. The DNA-A contains six open reading frames (ORFs): AC1, AC2, AC3, AC4, AV1, and AV2. These ORFs encode six proteins: AC1 (Replication initiator protein, Rep), AC2 (Transcriptional activator protein, TrAP), AC3 (Replication enhancer protein, Ren), AC4, AV1 (coat protein, CP), and AV2 (pre-coat protein). While, DNA-B has two ORFs, BC1 and BV1, in which BC1 encodes a movement protein and BV1 encodes a nuclear shuttle protein (NSP) (Moriones et al., 2017).

Managing begomoviruses, particularly in the field, presents significant challenges. Besides cultural management practices, the current strategies for the management of ToLCNDV include the management of whitefly vectors and the deployment of resistant varieties. However, vector control through insecticides leads to environmental pollution, insecticidal resistance and, poses risks to human health (Gill et al., 2014; Tudi et al., 2021; Juache-Villagrana et al., 2022). The development of resistant varieties relies on introducing six resistance loci, *Ty-1 to Ty-6* from wild relatives (Gill et al., 2019). A recent report showed a breakdown of *Ty-1-based* resistance at higher temperatures, indicating its less resilience under changed climatic conditions (Koeda and Kitawaki, 2023). Other than *Ty* gene-mediated resistance, limited efforts have been made to identify newer sources of resistance with different genes as such studies are time-consuming and labor-intensive. Recently, an R gene, *SlSw5a*, in the tomato cultivar H-88-78-1, was reported to provide resistance against ToLCNDV (Sharma et al., 2021). Besides these management options, RNAi-based transgenic and non-transgenic approaches have shown promising results. Various genes of begomoviruses, including AV1, AV2, and AC1, have been targeted for imparting resistance through RNAi-based methods (Mubin et al., 2007; Praveen et al., 2007; Singh et al., 2015, 2019). Developing artificial microRNAs (amiRs) that target the genes responsible for viral replication, transmission, and symptom development also presents a promising approach to limit the multiplication and spread of geminiviruses. Application of artificial miRNA designed against the AV1 (coat protein), AV2 (Pre-coat protein) (Vu et al., 2013) and AC1 (Rep protein), showed tolerance to ToLCNDV (Vu et al., 2013; Sharma and Prasad, 2020). Embrapa 5.1, a bean golden mosaic virus-resistant cultivar developed using RNAi approach, showed disease resistance in common beans (Faria et al., 2014). However, strict regulations in different countries hinder the deployment of transgenic crops in the field. Besides that, concerns from the public acceptance regarding the safety and environmental impact of genetically modified organisms (GMOs) further restrict the widespread adoption of transgenic approaches in agriculture. To harness the benefit of RNAi without involving the transgenic approach, an alternative means is the exogenous application of double-stranded RNA (dsRNA) molecules homologous to viral segments for conferring resistance against viruses in plants (Voloudakis et al., 2022). This approach is effective against various RNA viruses and viroids (Delgado-Martín et al., 2022; Gupta et al., 2021). However, very few reports are available on the success of the exogenous application of the dsRNA in the case of DNA viruses (Namgial et al., 2019). We reported earlier that dsRNA treatment targeting the AC2 gene of ToLCNDV significantly reduced viral titer in *Nicotiana benthamiana* plants (Sangwan et al., 2023). Recently, another study showed the effect of dsRNA against all the coding regions of the ToLCNDV DNA-A in zucchini (Frascati et al., 2024). However, the effect of topical dsRNA application against ToLCNDV in tomato, its natural host has not been studied.

In recent years, extensive research has been conducted into begomoviruses virulence and pathogenicity-associated proteins. Among the begomovirus-encoded proteins, different viral suppressors of RNA silencing proteins (VSRs) encoded by ORFs AC2, AC4 and AV2 interfere with the plant’s RNA silencing pathway and often act as symptom determinants (Roshan et al., 2018; Kulshreshtha et al., 2019; Zhai et al., 2022). In the case of ToLCNDV, NSP, coat protein, pre-coat protein and TrAP have been reported to act as pathogenicity determinants (Hussain et al., 2005; Basu et al., 2018; Vo et al., 2023). Earlier, we identified AV2, AC2 and AC4 genes of ToLCNDV function as VSRs (Sarkar et al., 2021). However, their involvement in viral pathogenesis and disease development remains to be understood. In this investigation, we aim to validate the involvement of suppressor proteins in determining the pathogenicity of ToLCNDV and to evaluate the efficacy of dsRNA targeting these genes in reducing viral infection in tomato plants. Through transient overexpression, followed by knockdown assays, we establish that all the VSRs encoded by ToLCNDV act as pathogenicity determinants. We also report topical dsRNA application against these pathogenicity determinants limits virus accumulation and attenuates symptom development in the model host plant, *N. benthamiana* and natural host plant, tomato. Thus, this study highlights the effectiveness of targeting pathogenicity determinants through a non-transgenic approach for managing ToLCNDV infection.

## Materials and methods

### Plant materials, virus construct, and whitefly culture

*Nicotiana benthamiana* and tomato seedlings were grown in an environmentally controlled growth chamber with 16 h light/8 h dark cycles (1,000–2,500 lux) at 24 ± 2°C and relative humidity of 70%. An agro-infectious construct of ToLCNDV, consisting of partial tandem repeats (PTR) of DNA-A (HQ141673) and DNA-B (HQ141674), was developed earlier in our laboratory (Jyothsna et al., 2013). These constructs were used together (mentioned as pToL in this article) for inoculation of ToLCNDV in the hpRNAi study. The symptomatic tomato plants that resulted from the inoculation of pToL alone were used as a source of virus culture for the whitefly inoculation study in dsRNA experiment. For whitefly inoculation, the aviruliferous whiteflies (biotype Asia II 1) were maintained on brinjal plants in an insect-rearing chamber at 28 ± 2°C, 50% relative humidity, 16 h light/8 h dark cycle, and 22,200 lux of illumination in an insect-free glass house.

### Development of overexpression, hairpin RNAi, and dsRNA constructs

Full-length AV2 (339 bp) (MW423574), AC2 (420 bp) (MW423576), and AC4 (177 bp) (MW423575) genes of ToLCNDV were previously amplified from ToLCNDV agroinoculated tomato plants using corresponding gene-specific primers and cloned into the pTZ57R/T vector. In this study, these genes were amplified with gene-specific primers (Supplementary Table S1) using Phusion™ High-Fidelity DNA Polymerase (2 U/μL) (Thermo Fisher Scientific, United States) and sub-cloned in to a plant overexpression vector with a GFP tag, pEarleyGate 103 (source: ABRC, stock CD3-685) using the *Xho*I restriction enzyme. The resulting constructs, OEAV2, OEAC2, and OEAC4, were then transformed into *E. coli* strain DH5 aLpha. Further, they were transformed into *Agrobacterium tumifaciens* strain GV3101. For the development of the hairpin RNAi constructs, the suppressor genes AV2, AC2, and AC4 were amplified (Supplementary Figure S1B) using three separate sets of primers (Supplementary Table S1) containing a *Bsa*I restriction site followed by a five-nucleotide sequence, which served as the fusion site of the inverted repeat of the pRNAiGG vector, which was used to generate hairpin RNAi constructs (hpRNAi) through Golden Gate assembly following recommended protocol (Supplementary Figure S1A; Yan et al., 2012). Briefly, the Golden Gate reaction was set up by combining 200 ng of PCR product, 200 ng of the pRNAiGG vector, 0.5 μL of T4 DNA ligase (5 U/μL) (Thermo Fisher Scientific, United States), 1 μL of 10X T4 DNA ligase buffer, and 0.5 μl of *Bsa*I enzyme (10 U/μL) (Thermo Fisher Scientific, United States). The resulting constructs, hpAV2, hpAC2, and hpAC4, were transformed into *E. coli* strain DH5 alpha, and positive hairpin constructs were confirmed by colony PCR using forward and reverse primers of the respective genes. Additionally, plasmid PCR was conducted using P21, P22, and respective reverse primers of the genes to confirm the proper orientation of the PCR amplicon (Supplementary Table S1; Supplementary Figure S1C). Confirmed hairpin constructs were then transformed into *A. tumifaciens* strain GV3101 for subsequent experimentation. Furthermore, the AV2, AC2, and AC4 genes were subcloned into the L4440 vector at *Xba*I and *Hind*III sites to produce double-stranded RNA (dsRNA). The L4440 vector contains two T7 polymerase promoters in sense and antisense orientations flanked by multiple cloning sites. Recombinant plasmids dsAV2, dsAC2, and dsAC4 were transformed into RNAse III deficient *E. coli* strain HT115 for dsRNA production.

### Agroinoculation

Single colonies from the agroculture of transformed OE, hpRNAi constructs, and pToL were grown overnight in a selective medium. The overnight culture was then used to prepare a fresh culture by growing it for 3–4 h. The bacterial cells were harvested and resuspended in an infiltration buffer (10 mM MES [pH 5.7], 10 mM MgCl2, and 150 μM acetosyringone) to attain an OD_600_ of approximately 0.5. This culture was incubated at room temperature in the dark for 2 h before infiltrating the leaves of 4-week-old *N. benthamiana* plants using a 2.0 mL needleless syringe. All the experiments were conducted in three independent replications with 5 plants in each replication. In the case of overexpression experiments, OE constructs were agroinfiltrated (1.5 mL of 0.5 OD culture per plant) and symptom expression was recorded daily and compared with empty vector pEarleyGate 103 infiltrated plants, which serves as negative control. To knock down the expression of the VSR transcripts, hpAV2/AC2/AC4 were first agroinfiltrated followed by agroinfiltration of corresponding overexpression constructs in the same leaf after two days. Similarly, to understand the effect of hairpin RNAi constructs on ToLCNDV infection, hpAV2, hpAC2, hpAC4 and their cocktail (hpCk) were agroinfiltrated (1.5 mL of 0.5 OD culture per plant) to the bottom portion near the petiole of leaves of *N. benthamiana* plant. Thereafter, agro-inoculation of pToL was performed two days post-hpRNAi construct inoculation at the apex of the same leaf. After agroinfiltration of the constructs, symptom development was recorded daily in the infiltrated plants. Based on the observed symptoms, they were classified into different grades (Supplementary Table S2).

### Reverse transcriptase PCR and confocal microscopy

To understand the systemic movement of transcripts of VSRs after inoculation of OE constructs, RNA was isolated from the systemic leaves using TRIzol reagent (Invitrogen). First strand cDNAs of all the VSRs were prepared gene-specific primers and RevertAid Reverse Transcriptase (200 U/μL) (Thermo Fisher Scientific, United States) following the manufacturer’s protocol. Thereafter, PCR amplification from the cDNA was done using gene-specific primers as mentioned earlier. After agroinoculation of the OE constructs, the expression of GFP-tagged VSRs in the systemic leaves was visualized after 48–60 h of inoculation, using Lieca Confocal Microscope PCS 5X. Systemic leaves of empty vector pEarleyGate 103 infiltrated plants have been taken as the negative control. The samples were observed under a GFP filter with excitation at 488 nm and emission at 500–550 nm.

### *In vivo* production of dsRNA

To attain a high yield of dsRNA, various parameters were optimized based on our previous work (Gupta et al., 2021). Transformed *E. coli* HT115 bacterial cells containing the respective constructs were induced with 1 mM IPTG for 4 h. Subsequently, the bacterial cells were collected through centrifugation at 6,000 × g for 10 min at 4°C. The cell pellet was resuspended in a 5 mL solution containing 1x PBS and 70% ethanol and incubated at 4°C for 30 min. After incubation, the cells were again collected through centrifugation and resuspended in 2.0 mL of 150 mM NaCl, followed by another incubation at 4°C for 1 h. The suspension was then again centrifuged, separating the dsRNA and other nucleic acids into a supernatant collected in a clean centrifuge tube. To understand the isolated dsRNA’s double-stranded nature, the supernatant was processed with RNAase-free DNase I and RNase in low and high salt concentrations (Libonati and Sorrentino, 1992). The purified dsRNA of AV2 (dsAV2), AC2 (dsAC2), and AC4 (dsAC4) was resolved in agarose gel electrophoresis to assess their quality.

### Exogenous application of dsRNA in *Nicotiana benthamiana* and tomato plants and evaluation of their efficacy against ToLCNDV

To prepare the dsRNA for exogenous application, 10 μg each of dsAV2, dsAC2, dsAC4 and dsCk (containing 3.33 μg dsAV2 + 3.33 μg dsAC2 + 3.33 μg dsAC4) were suspended separately in 100 μL deionized water having 0.01% celite. Initially, to test the efficacy of dsRNA, dsAC4 was gently rubbed on the adaxial surface of the leaves of three leaves 21-day-old seedlings of *N. benthamiana.* In case of tomato, all the dsRNA preparations were applied to the 14-day-old tomato plants. In the dsRNA-treated plants, ToLCNDV inoculation was carried out simultaneously through agro-infiltration of pToL (in *N. benthamiana* plants) or whitefly inoculation (in tomato plants). For whitefly transmission experiment, plants of tomato cv. Pusa Ruby were inoculated with pToL construct. After the appearance of symptoms in the pToL inoculated plants, presence of the virus was confirmed by PCR and such plants were used as a source of inoculum for whitefly transmission. The aviruliferous whiteflies were allowed to feed on ToLCNDV-infected symptomatic tomato plants (obtained through inoculation of pToL) for 24 h to acquire the virus. Each dsRNA-treated plant was then exposed to six viruliferous whiteflies for 16–20 h. Subsequently, the whiteflies were eliminated by spraying a contact insecticide, acephate (75 SP), with a recommended dose (1–1.5 g/L). The plants were then observed for disease development for 30 days.

### Quantification through real-time PCR

A quantitative real-time PCR analysis was performed to quantify the knockdown of VSR transcripts by hpRNAi constructs. For this purpose, RNA was isolated and cDNA was prepared for each VSR transcript as described earlier from the inoculated and systemic leaves on 4 days post inoculation (dpi). A quantitative PCR was also performed to determine the relative viral load in the plants treated with hpRNAi and dsRNA constructs. In the context of hairpin experiments, the analysis of results was done on the 4 and 11 dpi (both systemic and local), while for dsRNA experiments viral load were assessed on the 14 and 28 dpi. Quantitative PCR (qPCR) was performed using the 1× KAPA SYBR® FAST qPCR Master Mix (Roche, F. Hoffmann-La Roche Ltd., Switzerland) and the CFX96 Touch^™^ Real-Time PCR detection system (Bio-Rad, Hercules, CA, USA). The Actin gene was selected as the reference control for the qPCR study. The primers used are listed in Supplementary Table S1. The relative reduction of viral load in both treated and untreated plants was calculated following the double Ct method, as described by Livak and Schmittgen (2001), keeping the viral load in untreated plants as 1 for comparison.

### Statistical analysis

To determine the statistical significance of plants treated with hpAV2, hpAC2, hpAC4 and hpCk from the control plants (only virus inoculated) on the height of the plant and the number of leaves that showed symptoms, a one- or two-way ANOVA followed by Tukey’s test was conducted using R software (version 4.3.1) (R Core Team, 2024). The Figures were plotted using the “ggplot2” package of R software. To determine the statistical significance of the various hairpin and dsRNA experiments, a student’s *t*-test was carried out (Student, 1908).

## Results

### AV2, AC2, and AC4 genes of ToLCNDV are genomic determinants of pathogenicity

**Figure 1:**
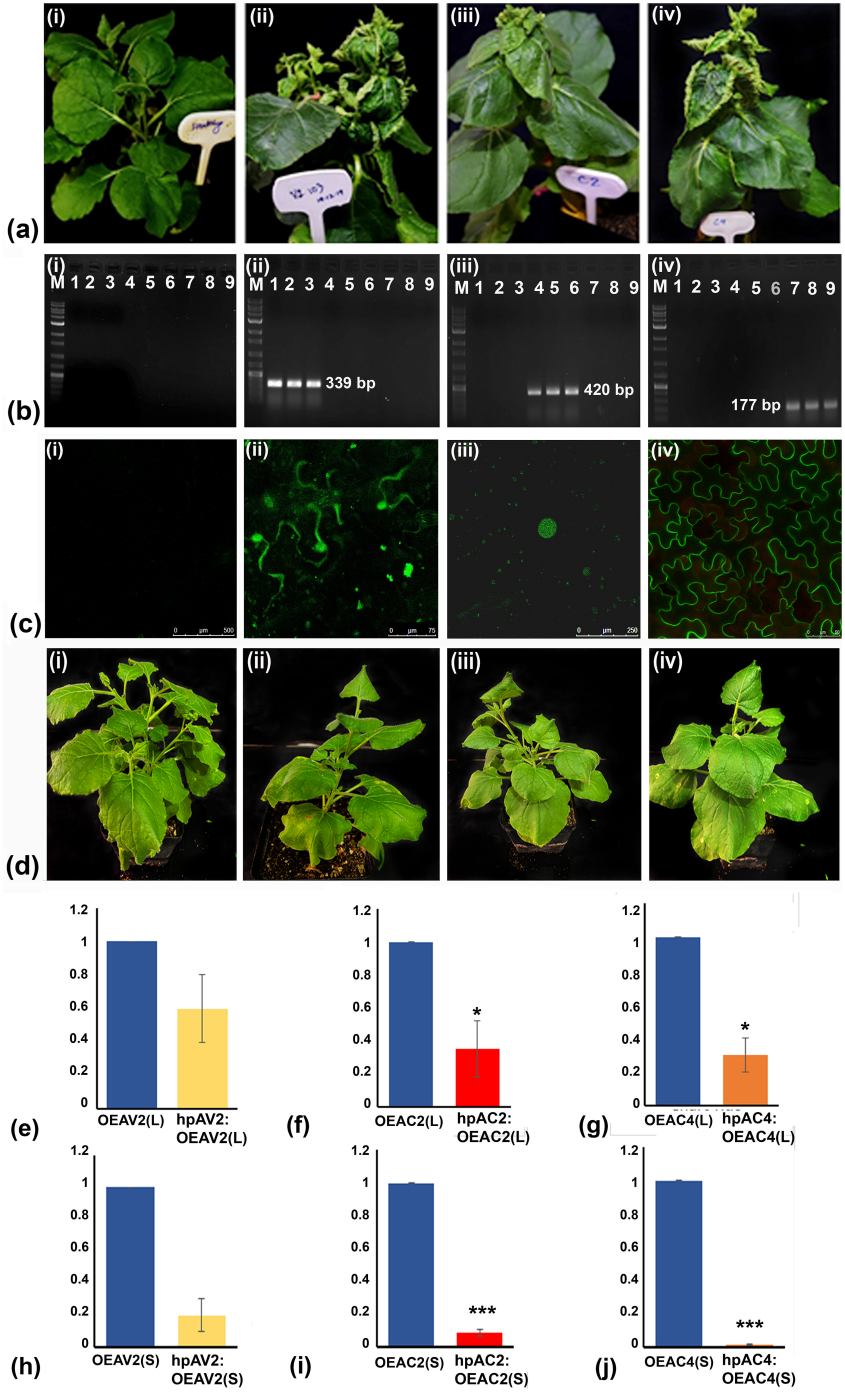
Pathogenicity determination of the three silencing suppressors encoded by DNA-A of ToLCNDV through transient overexpression and knockdown assay in *Nicotiana benthamiana* plants. **(A)** Symptom induction by AV2, AC2, and AC4 was determined via agroinfiltrating their respective GFP-tagged overexpression constructs (OE). Five plants were infiltrated for each construct. No symptom was observed in empty vector (pEarleyGate 103) infiltrated plants (i), severe curling of leaves, enation, and vein thickening were observed in plants infiltrated with OEAV2 (ii), downward curling of leaves in plants infiltrated with OEAC2 (iii), and curling of margin of the leaves and enation in plants infiltrated with OEAC4 (iv). **(B)** Transcripts of all three silencing suppressor genes were detected in the systemic leaves of their respective OE construct infiltrated plants (ii–iv). The plants inoculated with empty vector, pEarleyGate 103 (negative control) did not yield any amplification (i). **(C)** Determination of expression of the respective GFP-tagged OE constructs in the systemic leaves through confocal microscopy showing subcellular localization of AV2 protein in the host cell membrane and nucleus (ii), AC2 protein in the nucleus (iii) and the AC4 protein in the host cell membrane (iv). As expected, GFP expression was not observed in systemic leaves of plants inoculated with empty vector, pEarleyGate 103 (negative control) (i). **(D)** Knockdown of AV2, AC2, AC4 transcripts in *N. benthamiana* plants infiltrated with hairpin RNAi (hpRNAi) followed by OE constructs (represented as hpAV2:OEAV2; hpAC2:OEAC2; hpAC4:OEAC4), showed relative reduction of symptoms in all the hpRNAi treated plants (ii-iv), while no phenotypic changes were observed in empty vector infiltrated plants (i). **(E–J)** Quantitative real-time PCR showed a significant reduction of transcripts of AV2, AC2, and AC4in local and systemic leaves after hpRNAi treatment. Student’s *t*-test was used to determine the significance of differences. *p < 0.05, **p < 0.01, ***p < 0.001. M, Molecular Marker.

Transient overexpression of individual viral suppressor of RNA silencing (VSR) of ToLCNDV on *N. benthamiana* leaf successfully produced the leaf curl symptom phenotype in the systemic leaves with different degrees and with different incubation times for three independent experiments. On average 80% of OEAV2-inoculated plants showed severe leaf curling, enation, and vein thickening symptoms within 12 dpi, while 73% of plants inoculated with OEAC2 exhibited downward curling symptoms after 30 days. In the case of OEAC4 inoculated plants, marginal curling and enation were observed after 22 days in 80% of inoculated plants. These observations suggest that all VSRs of ToLCNDV are symptom determinants. Among these, the VSR AV2 showed the maximum ability to trigger disease symptoms in many plants (Figure 1A; Table 1). Typical leaf curl symptoms in the systemic leaves prompted us to confirm the presence of VSR transcript through reverse transcriptase PCR. Analysis revealed amplification of transcripts of the respective VSRs in the distal leaves (AV2-339 bp; AC2-420 bp; AC4-177 bp), affirming the systemic movement of the RNA transcripts of VSRs in the inoculated plants (Figure 1B). Amplification of the backbone region of pEarleyGate 103 vector was carried out from the systemic leaves, which did not yield any results (Data not shown), which overrule the amplification of VSR transcript due to either the passive movement of the agrobacteria or systemic movement of the backbone plasmids. The confocal imaging of the VSR proteins fused with GFP was also detected in the systemic leaves (Figure 1C). While AC2 and AC4 proteins were found to be localized in the nucleus and cell membrane, respectively, AV2 was present in both the nucleus and cell membrane. This indicates that the VSRs of ToLCNDV could move systemically, express their proteins, and produce symptoms in distal leaves (Figure 1B).

**Table 1:** Pathogenicity determination through overexpression and knockdown of AV2, AC2, and AC4 genes of ToLCNDV in systemic leaves of *Nicotiana benthamiana* plants.

Treatment	Type of symptom	Average time interval for appearance of symptom (in days)	Mean percentage of plants showing symptoms (%)
pEarleyGate 103 (negative control)	No symptom (Grade 0)	-	-
OEAV2	Severe curling of leaves, enation, vein thickening, smelling of leaves (Grade 3)	12	80
OEAV2: hpAV2	Mild curling of leaves (Grade 1)	20	50
OEAC2	Downward curling (Grade 2)	20	73
OEAC2: hpAC2	Mild curling of leaves (Grade 1)	27	26
OEAC4	Curling of margin of leaves (Grade 2)	22	80
OEAC4: hpAC4	Mild curling of leaves (Grade 1)	30	20

Knockdown of the VSRs in the transiently overexpressed *N. benthamiana* plants through hpRNAi constructs delayed the onset of symptoms and severity. Plants treated with OEAV2:hpAV2, and OEAC2:hpAC2, showed mild leaf curling symptoms at 20 and 27 dpi, respectively. However, in OEAC4:hpAC4 inoculated plants no symptom was observed up to 30 days (Figure 1D; Table 1). The reduction in disease severity and delay in symptom development of the individual VSRs after hpRNAi treatment is correlated with the real-time PCR result of VSR transcript accumulation in both the local (L) and the systemic (S) leaves. Notably, the degree of inhibition was higher in the systemic leaves compared to the local leaves. Among the various treatments, maximum transcript reduction was observed in the plants treated with OEAC4:hpAC4 with 70 and 99% reduction in the local and systemic leaves, respectively. Plants infiltrated with OEAC2:hpAC2 showed a moderate reduction, reflecting 65 and 91% in the locally treated and systemic leaves, respectively. However, the least reduction of transcript (40 and 80% in the locally treated and systemic leaves, respectively) was observed in OEAV2:hpAV2 inoculated plants (Figures 1E–J). Thus, both overexpression and targeted knockdown experiments confirm the involvement of AV2, AC2, and AC4 in pathogenicity.

### Hairpin RNAi construct developed against AC4 ORF of ToLCNDV resulted in maximum reduction of symptoms upon virus inoculation in *Nicotiana benthamiana*

**Figure 2:**
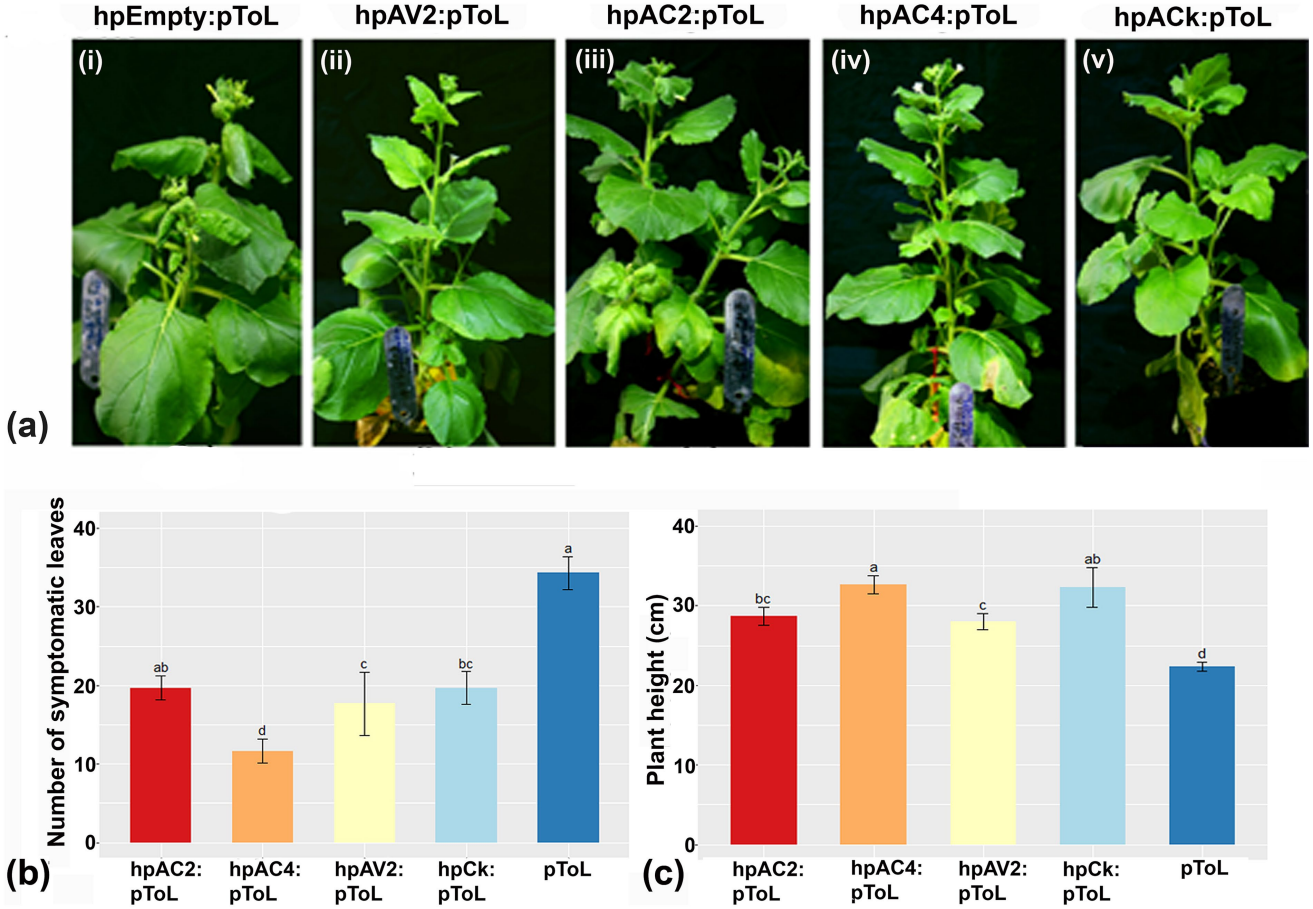
Assessment of the efficacy of hpRNAi constructs hpAV2, hpAC2 and hpAC4 after challenge inoculation with infectious construct of ToLCNDV (pToL) in *N. benthamiana* plants. Photos were taken after 30 days of virus inoculation. **(A)** hpRNAi constructs of AV2, AC2 and AC4 genes were agroinfiltrated separately or together onto the bottom portion of *N. benthamiana* leaves. pToL construct was infiltrated after 2dpi of hpRNAi inoculation at the top portion of the same hpRNAi-inoculated leaves. All hpRNAi treated plants showed reduced symptoms (ii-v). In case of negative control, an empty RNAi-GG vector was infiltrated followed by pToL infiltration which exhibited severe downward curling (i). **(B,C)** Effect of hpRNAi on disease incidence and plant height, respectively after 45 days of virus inoculation. Similar letters above bars are not significantly different at *p* < 0.05 using one- or two-way ANOVA followed by Tukey’s test.

**Figure 3:**
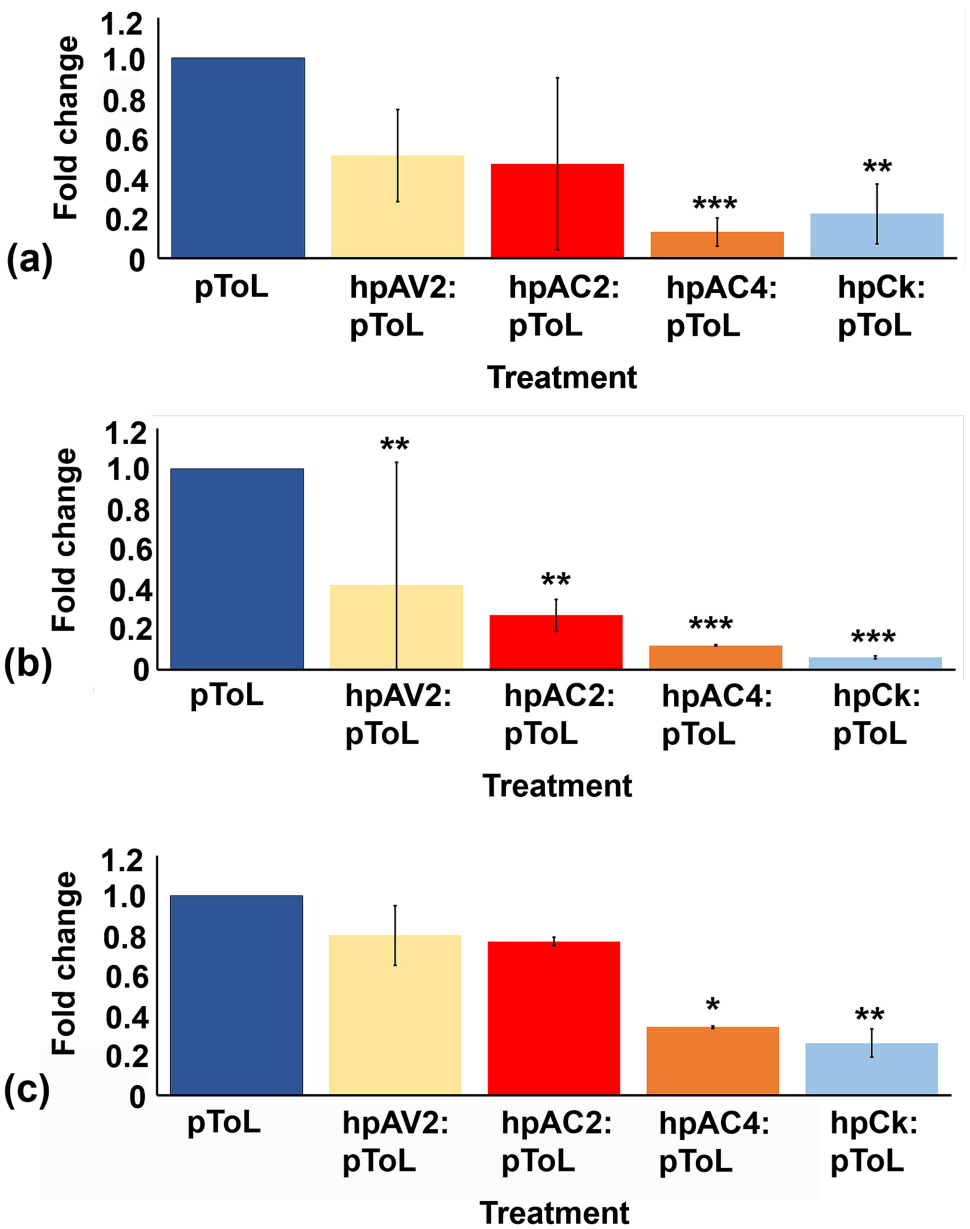
Quantitative real-time PCR assay to determine the relative reduction of ToLCNDV accumulation in hpRNAi-treated *N. benthamiana* plants at different time intervals in infiltrated and systemic leaves. **(A)** infiltrated leaves at 4 dpi, **(B)** infiltrated leaves at 12 dpi, and **(C)** systemic leaves at 12 dpi. Actin was used as an endogenous control. Student’s *t*-test was used to determine the significance of differences. *p < 0.05, **p < 0.01, ***p < 0.001.

The reduction of individual VSRs transcript through respective hairpin constructs prompted us to evaluate the effectiveness of these hp. constructs against ToLCNDV viral load in *N. benthamiana* plants. Plants treated with hpAC4 and hpCk exhibited the most significant reduction in symptoms, with no observed symptoms up to 30 dpi with pToL (Grade 0), while hpAV2 and hpAC2 treated ones showed mild leaf curling after 15 dpi (Grade 1) (Figure 2A; Supplementary Table S2). This indicates that the transient expression of hairpin RNAi constructs targeting VSR effectively limits symptom development of ToLCNDV. Additionally, we examined the impact of hairpin constructs on the occurrence of symptomatic leaves and plant height. We observed a significant difference in the number of symptomatic leaves among plants treated with hpAC4, hpAV2, and hpCk (Figure 2B). Likewise, there was a notable variation in plant height in plants treated with hpAC2, hpAV2, hpAC4, hpCk, and the control plants (only pToL inoculated) (Figure 2C). Furthermore, real-time PCR analysis conducted at 4 dpi on treated leaves and 12 dpi on local and systemic leaves showed a reduction in the viral load compared to control plants that were inoculated solely with pToL. At 4 dpi, plants treated with hpAV2, hpAC2, hpAC4, and hpCk exhibited a 49, 53, 87 and 78% reduction in viral titer, respectively (Figure 3A). Results from the localized tissue at 12 dpi showed a reduction of 58, 73, 88, and 94%, respectively (Figure 3B), while in the case of systemic leaves, there was a reduction of 20, 23, 66 and 74%, respectively (Figure 3C).

### Exogenous application of dsRNA against the pathogenicity determinants of ToLCNDV limits symptom development and virus load in tomato plants

**Figure 4:**
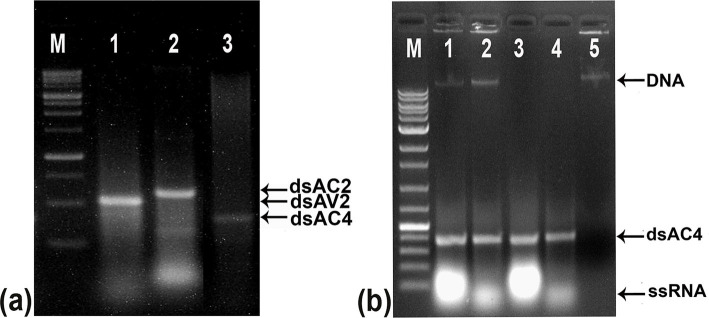
Agarose gel electrophoresis of purified dsRNA from *E. coli* strain HT115 and confirming the double-stranded nature through nuclease treatment. **(A)** Purified dsRNA from bacterial culture Lane 1: dsAV2 (479 bp), Lane 2: dsAC2 (560 bp), Lane 3: dsAC4 (317 bp). The increase in the size of purified dsRNA is due to the flanking region between T7 promoters. **(B)** Agarose gel showing nuclease digestion of the isolated dsRNA from the bacteria transformed with pL4440. Lane 1: untreated dsAC4, Lane 2: RNase A in high salt resisted digestion of 317 bp fragment, Lane 3: DNase treated dsAC4, digested DNA but no effect on dsRNA, Lane 4: both DNase and RNase treatment at high salt, Lane 5: completely digested dsAC4 in low salt condition, indicating double-stranded form. M, Molecular Marker.

**Figure 5:**
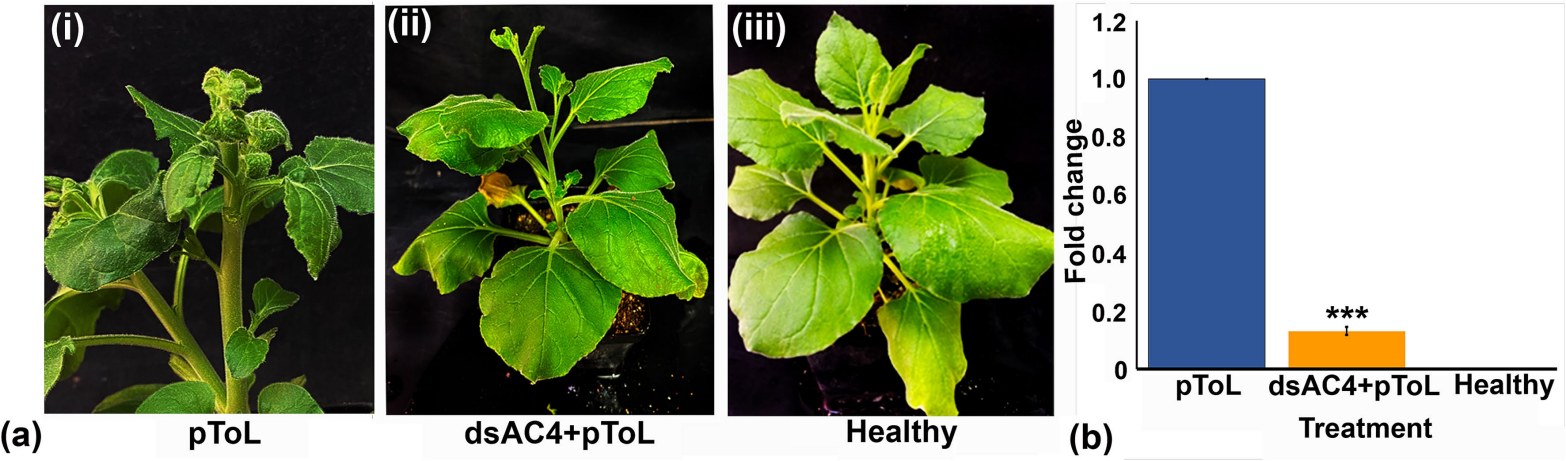
Effect of topically applied dsAC4 in *N. benthamiana* plants infiltrated with pToL construct based on symptom phenotypes and viral load estimation through qPCR. **(A)** Symptom phenotype observed in *N. benthamiana* plants after pToL infiltration at 11 dpi (i), simultaneous infiltration of dsAC4 and pToL (ii) and MES buffer inoculation (mock) (iii). dsAC4 treated plants showed reduction in symptom after virus inoculation. **(B)** Quantitative real-time PCR showing significant relative reduction of viral titer in dsAC4 treated plants compared to pToL infiltrated plant. Actin was used as an endogenous control. Student’s *t*-test was used to determine the significance of differences. *p < 0.05, **p < 0.01, ***p < 0.001.

**Figure 6:**
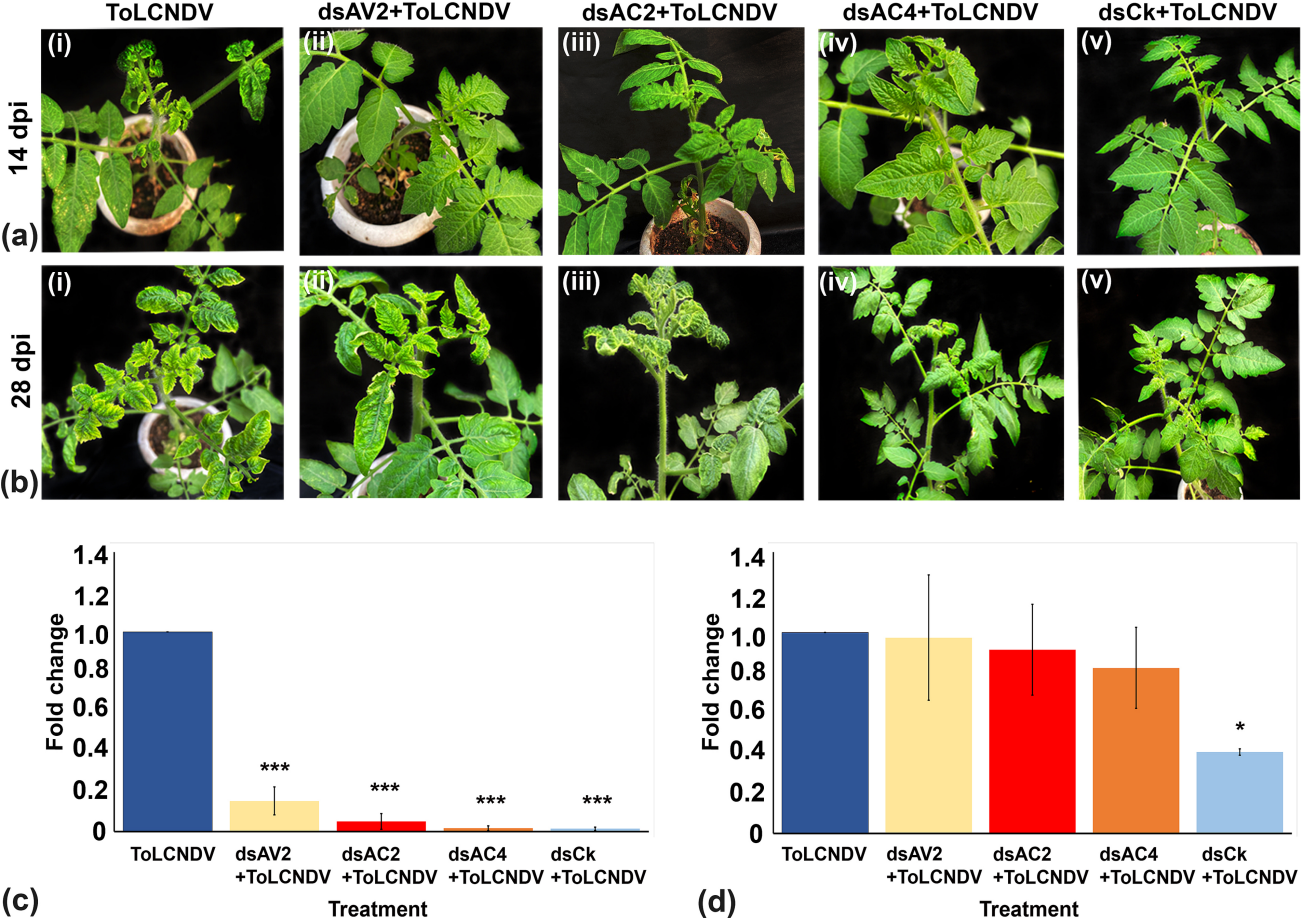
Effect of topically applied dsRNA on ToLCNDV infection in tomato plants at different time intervals. **(A)** At 14 dpi, leaf curl symptom was observed in control plants inoculated with ToLCNDV through whitefly transmission (I), while at 14 dpi, plants treated with dsAV2, dsAC2, dsAC4 and dsCk did not exhibit any symptoms (ii–v). **(B)** At 28 dpi, severe symptoms were observed in control plants (ToLCNDV inoculated) (i), mild symptoms were observed at 28 dpi in the dsAV2 and dsAC2-treated ones (ii–iii), while in dsAC4 and dsCk treated plants remains symptom free (iv–v). **(C,D)** Quantitative real-time transcriptase PCR (qRT-PCR) showing significant reduction of viral titer at 14 and 28 dpi. dsCk treatment showed the best results followed by dsAC4. Actin was used as an endogenous control. Student’s *t*-test was used to determine the significance of differences. *p < 0.05, **p < 0.01, ***p < 0.001.

dsRNA against AV2 (dsAV2: 479 bp), AC2 (dsAC2: 560 bp), and AC4 (dsAC4: 317 bp) genes of ToLCNDV were produced and purified from *E. coli* strain HT115 (Figure 4A). RNase A treatment at low salt concentrations, completely degraded the purified RNA produced against AC4 gene. At the same time, it remained intact at high salt concentrations, thus confirming the double-stranded nature of the purified RNA (Figure 4B). The reduction of virus accumulation in hpAC4-treated *N. benthamiana* plants prompted us to ascertain the impact of exogenous application of dsAC4 against ToLCNDV in *N. benthamiana* plants. dsAC4-treated plants inoculated with pToL exhibited a reduction in symptom severity and a delay in the onset of symptoms compared to plants only inoculated with the virus (Figure 5A). Further, real-time PCR analysis at 11 dpi showed an 87% reduction in viral load in dsAC4-treated *N. benthamiana* plants compared to the untreated ones (Figure 5B). Further, we investigated the effect of exogenous dsRNAs (dsAV2, dsAC2, dsAC4, and dsCk) on ToLCNDV infection in tomato plants (natural host) at 14 and 28 dpi. dsAV2, dsAC2, dsAC4, and dsCk treated plants remained symptom-free whereas untreated tomato plants exhibited leaf curling symptoms in young leaves at 14 dpi (Figure 6A). A similar trend was also observed in real-time PCR analysis which revealed that the viral load was reduced in all dsRNA-treated plants, where the maximum reduction was found in dsCk (99%) followed by dsAC4 (98%), dsAC2 (95%), and dsAV2 (84%) (Figure 6C). However, symptoms started appearing in different frequencies at different time points in the dsRNA-treated plants. Of all the dsRNA treatments, dsCk and dsAC4 exhibited the lowest number of symptomatic plants (11%) and also showed the maximum delay in the onset of symptoms (26 and 22 days respectively) (Figure 6B; Table 2). The real-time PCR analysis at 28 dpi revealed that the dsCk treated plants exhibited the most significant reduction (60%) in viral load, followed by the dsAC4 (18%) treated plants (Figure 6D). Thus, dsCK and dsAC4 treatment in both *N. benthamiana* and tomato plants showed maximum protection against ToLCNDV.

**Table 2:** Effect of dsRNA treatment on symptom onset on leaves of tomato plants after whitefly transmission of ToLCNDV.

Treatment	Time interval for appearance of symptom (in days)	Mean percentage of plants showing symptoms (%)
ToLCNDV (control)	14	100%
dsAC2 + pToL	16	47%
dsAV2 + pToL	20	23%
dsAC4 + pToL	22	11%
dsCk+pToL	26	11%

## Discussion

In this study, we investigated the role of VSRs encoded by ToLCNDV DNA-A in symptom development through transient overexpression followed by knockdown of individual VSR transcripts in model plant, *N. benthamiana*. Furthermore, we examined the effects of topical application of dsRNA, targeting the VSRs of ToLCNDV on virus titer and symptom development in its natural host plant, tomato. Upon transient overexpression of the OE constructs of VSRs *viz.* OEAV2, OEAC2 and OEAC4, typical symptoms were observed in systemic leaves of *N. benthamiana*, indicating that they act as symptom determinants, with the AV2 protein showing the maximum ability to induce disease symptoms in more plants. In earlier studies, the coat protein gene, pre-coat protein, TrAP and NSP of ToLCNDV were shown to be responsible for pathogenicity (Hussain et al., 2005; Basu et al., 2018; Vo et al., 2023). The VSRs of other begomoviruses, like the AV2 gene (from Potato virus X-based vector) of tomato leaf curl Palampur virus (ToLCPalV), AC5 gene of mungbean yellow mosaic India virus (MYMIV), V2, C2 and C4 of croton yellow vein mosaic virus (CYVMV) were also reported to be symptom determinants (Li et al., 2015; Roshan et al., 2018; Zhai et al., 2022).

Detection of the RNA transcripts of VSRs through Reverse transcriptase PCR (RT-PCR) and observation of GFP-tagged VSR proteins through confocal microscopy in the systemic leaves supports our hypothesis that the VSR transcripts can move systemically and express their proteins. The localization of the three proteins aligns with our previous finding on the subcellular localization of VSRs of ToLCNDV (Sarkar et al., 2021). Such systemic movement of VSR transcripts has been shown earlier in the case of croton yellow vein mosaic virus (Zhai et al., 2022). Systemic movement of small and long coding and non-coding RNAs has been documented by many researchers (Brosnan et al., 2007; Biedenkopf et al., 2020; Cheng et al., 2023). Furthermore, co-expression of ToLCNDV VSRs and their respective hairpin constructs showed a reduced accumulation of VSR transcripts in both inoculated and systemic leaves, which resulted in delayed symptom expression with less disease incidence and severity. The effect of VSR knockdown was more pronounced in the systemic leaves than in the treated ones. This may suggest the movement of fewer VSR transcripts, as most of them were degraded in the treated leaves. Thus, overexpression and knockdown assays conclusively revealed the critical role of VSRs in symptom development. In the case of ToLCPalV, AC4 protein was shown to be acting as a pathogenicity determinant as a mutation in N-terminal glycine residue abolished pathogenicity (Kulshreshtha et al., 2019). However, the precise mechanism by which these VSRs (encoded by ToLCNDV) induce symptoms remains to be investigated in the future.

These findings prompted us to investigate the feasibility of targeting VSRs for plant virus management. Previous studies have shown that RNAi-mediated transgenic resistance can effectively provide resistance against begomoviruses. For instance, hairpin RNA designed against the AC2 gene of MYMV and the AV2 gene of tomato yellow leaf curl virus-Oman is effective in blocking viral accumulation in transgenic conditions (Ammara et al., 2015; Shanmugapriya et al., 2015). In addition to these, the use of artificial trans-acting small interfering RNA (tasiRNA) designed against AC2 and AC4 genes (Singh et al., 2015) and antisense RNA targeting the AV1 and AV2 genes of ToLCNDV had been demonstrated to reduce the viral load in transgenic plants (Mubin et al., 2007; Singh et al., 2015). However, concerns over the potential negative effects of genetically modified plants on human health and the environment and their limited acceptance by consumers highlight the need to explore alternative non-transgenic strategies. In this study, we evaluated the efficacy of hpRNAi constructs targeting the VSRs of ToLCNDV upon virus inoculation in *N. benthamiana* plants under transient conditions. Interestingly, the knockdown was more pronounced in locally treated leaves than systemic ones. A possible explanation could be the rapid replication and spread of the virus in contrast to the time it takes for the silencing signal to move, which occurs over several days (Voinnet et al., 1998). All three hpRNAi constructs partially target their overlapping transcripts and cumulatively reduce the viral load. However, hpAC4 showed the maximum reduction, probably because it indirectly targets the transcript of *the* AC1 gene (involved in the initiation of replication) and thus hinders the replication of the viral genome. The selection of genes is crucial for effective virus management through dsRNA-mediated approach. Numerous studies have indicated the promising effects of exogenous dsRNA against RNA viruses (Mitter et al., 2017; Kaldis et al., 2018; Tabein et al., 2020; Vadlamudi et al., 2020; Gupta et al., 2021; Holeva et al., 2021; Delgado-Martín et al., 2022). However, limited literature is available on the application of dsRNA against DNA viruses (Washington et al., 2021). In this study, as hpAC4 showed the highest efficacy, we investigated the effect of exogenous application of dsAC4 against TolCNDV in the model host plant, *N. benthamiana*, which resulted in a significant decrease in viral load and symptom expression. As the RNAi protection pattern could differ between the model and natural host plants the effect of dsRNA against all the symptom determinants of ToLCNDV was studied in natural host plant, tomato. Notably, dsCk followed by dsAC4 provided the highest level of protection against ToLCNDV. Our previous study also showed that a cocktail of dsRNA combining dsAC2, dsAV2, and dsAC4 against ChiLCV significantly reduces the virus load and delayed symptom development (Washington et al., 2021). The results are consistent with the outcomes reported in a recently published study, which showed a decrease in the occurrence of infected plants and a delay in the appearance of symptoms associated with ToLCNDV in dsRNA-treated zucchini plants (Frascati et al., 2024).

Based on our current understanding, we reconfirm that the VSRs encoded by the DNA-A component of ToLCNDV serve as symptom determinants. In addition, we have also demonstrated the effectiveness of exogenous dsRNA, which targets the symptom determinants of ToLCNDV, in reducing viral titer and limiting symptom development in its natural host plant, tomato. Nevertheless, the siRNA produced from the hpRNAi constructs and dsRNA constructs may have partially targeted their overlapping ORF, providing an additional benefit. Therefore, our research contributes to the existing body of knowledge by studying the effects of exogenous dsRNA against DNA viruses, which complements the extensive research that has been conducted against RNA viruses.

## Data Availability

The datasets presented in this study can be found in online repositories. The names of the repository/repositories and accession number(s) can be found in the article/Supplementary material.
