# Supplementary material for: Identification of pathogenicity determinants in ToLCNDV and their RNAi-based knockdown for disease management in Nicotiana benthamiana and tomato plants
Source: Front Microbiol. 2024 Nov 27;15:1481523. doi: 10.3389/fmicb.2024.1481523 (PMC11631908; doi:10.3389/fmicb.2024.1481523)
Supplement: Supplementary file 1 [file Data_Sheet_1.ZIP › Revised_Supplementary files/01_Figure S1.docx]

**
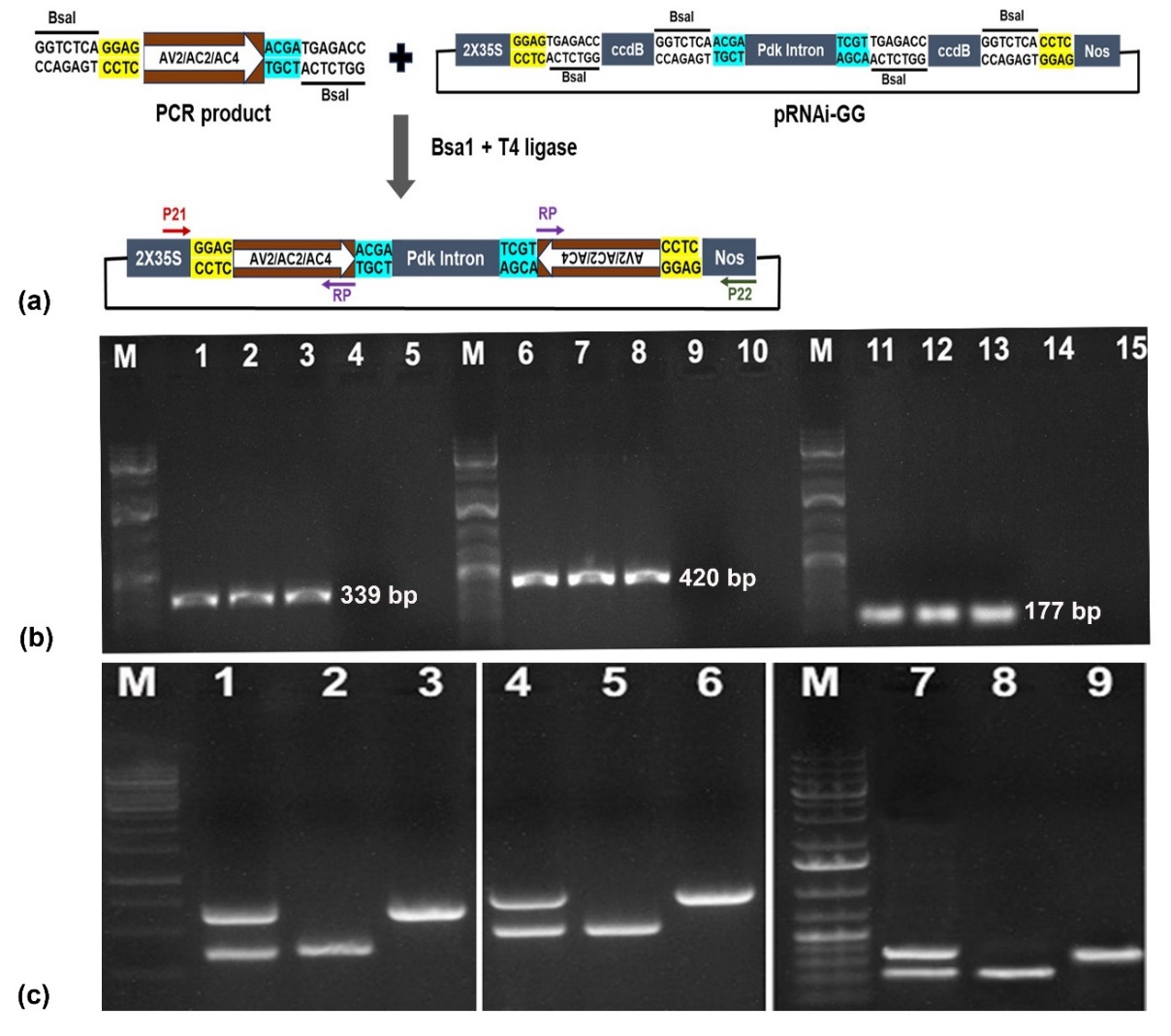
**

**Figure S1** Development of hairpin RNAi constructs using Golden Gate assembly. (a) Schematic representation of the development of hairpin RNAi constructs against suppressor genes of ToLCNDV. (b) Agarose gel electrophoresis of PCR amplicon of AV2, AC2 and AC4 genes of ToLCNDV using primers flanked with compatible linker to pRNAi-GG Golden Gate vector. Lane 1-3: PCR amplified products of AV2 (339 bp); Lane 6-8: PCR amplified products of AC2 (420 bp); Lane 11-13: PCR amplified products of AC4 (177 bp); Lane 4,9,14: Empty vector control; Lane 5,10,15: reagent control. (c) Agarose gel electrophoresis of PCR amplicons from pRNAi-GG vector harbouring intron-containing inverted arms of AV2, AC2 and AC4 genes of ToLCNDV using p21, p22 and reverse primer of each gene. Lane 1,4,7: PCR product using p21, p22 and reverse primer of AV2/AC2/AC4 genes showed the presence of two inverted arms of corresponding genes with intronic sequence. Lane 2,5,8: PCR product using p21 and reverse primer of AV2/AC2/AC4 genes showed the presence of one side of the inverted fragments of corresponding genes. Lane 3,6,9: PCR product using p22 and reverse primer of AV2/AC2/AC4 genes showed the presence of the second side of two inverted fragments of corresponding genes. M: Molecular Marker
