# Supplementary material for: Identification of pathogenicity determinants in ToLCNDV and their RNAi-based knockdown for disease management in Nicotiana benthamiana and tomato plants
Source: Front Microbiol. 2024 Nov 27;15:1481523. doi: 10.3389/fmicb.2024.1481523 (PMC11631908; doi:10.3389/fmicb.2024.1481523)
Supplement: Supplementary file 1 [file Data_Sheet_1.ZIP › Revised_Supplementary files/02_Table S1_and_S2.DOCX]

**Table S1** List of different primers used in the study

| **Name** | **Primer sequence (5’-3’)** | **Tm (^0^C)** | **Expected amplicon size (bp)** | **Purpose** |
| --- | --- | --- | --- | --- |
| AV2-XhoI F | TAACTCGAGATGTGGGATCCATTATTGCA | 59.0 | 339 | Cloning of AV2 in pEarlyGate103 |
| AV2-XhoI R | TAACTCGAGTACATTCTGTACATTCTGGGT |  |  |  |
| AC2-XhoI F | TAACTCGAG ATGCAGTCTTCATCACACTC | 66.0 | 420 | Cloning of AC2 in pEarlyGate103 |
| AC2-XhoI R | TAACTCGAG AGGACCTGGGTTTTGAAGACTCTC |  |  |  |
| AC4-XhoI F | TAACTCGAG ATGGGTCTCCGCATATCCATG | 64.0 | 177 | Cloning of AC4 in pEarlyGate103 |
| AC4-XhoI R | TAACTCGAG CGAACGTCTCCATCTTTGTC |  |  |  |
| AV2-RNAiGG-F | ACCAGGTCTCAGGAGATGTGGGATCCATTATTGCA | 64.0 | 339 | Amplification of AV2 region using linker flanked primers for hairpin RNAi construct |
| AV2-RNAiGG-R | ACCAGGTCTCATCGTTACATTCTGTACATTCTGGGT |  |  |  |
| AC2-RNAiGG-F | ACCAGGTCTCAGGAGATGCAGTCTTCATCACAC | 64.8 | 420 | Amplification of AC2 region using linker flanked primers for hairpin RNAi construct |
| AC2-RNAiGG-R | ACCAGGTCTCATCGTAGGACCTGGGTTTTGAAG |  |  |  |
| AC4-RNAiGG-F | ACCAGGTCTCAGGAGATGGGTCTCCGCATATCC | 65.0 | 177 | Amplification of AC4 region using linker flanked primers for hairpin RNAi construct |
| AC4-RNAiGG-R | ACCAGGTCTC GAACGTCTCCATCTTTGTC |  |  |  |
| P21-RNAiGG-F | ACCATTTACGAACGATAGCC | 54.0 | Size depends upon reverse primer of gene | To confirm the inverted arm of hairpin construct |
| P22-RNAiGG-R | GTAAAACGACGGCCAGTG | 54.0 | Size depends upon reverse primer of gene | To confirm the inverted arm of hairpin construct |
| BM-843 | GGTATTGTGTTGGACTCGGG |  | 200 bp | Partial actin gene used as a reference gene |
| B-850 | GCTGTGGTAGTGGATGAGTAAC |  |  |  |

**Table S2** Symptom phenotype with corresponding symptom grade to quantify the leaf curl disease in tomato after inoculation of ToLCNDV

| **Symptom phenotype** | **Grade** |
| --- | --- |
| No symptom | 0 |
| Mild curling of leaves | 1 |
| Downward/ upward/ marginal curling of leaves | 2 |
| Severe curling of leaves, enation, vein thickening, smalling of leaves | 3 |
