# Supplementary material for: Identification of pathogenicity determinants in ToLCNDV and their RNAi-based knockdown for disease management in Nicotiana benthamiana and tomato plants
Source: Front Microbiol. 2024 Nov 27;15:1481523. doi: 10.3389/fmicb.2024.1481523 (PMC11631908; doi:10.3389/fmicb.2024.1481523)
Supplement: Supplementary file 1 [file Data_Sheet_1.ZIP › Revised_Supplementary files/03_Original_Gel_images_revised.pptx]

## Slide 1
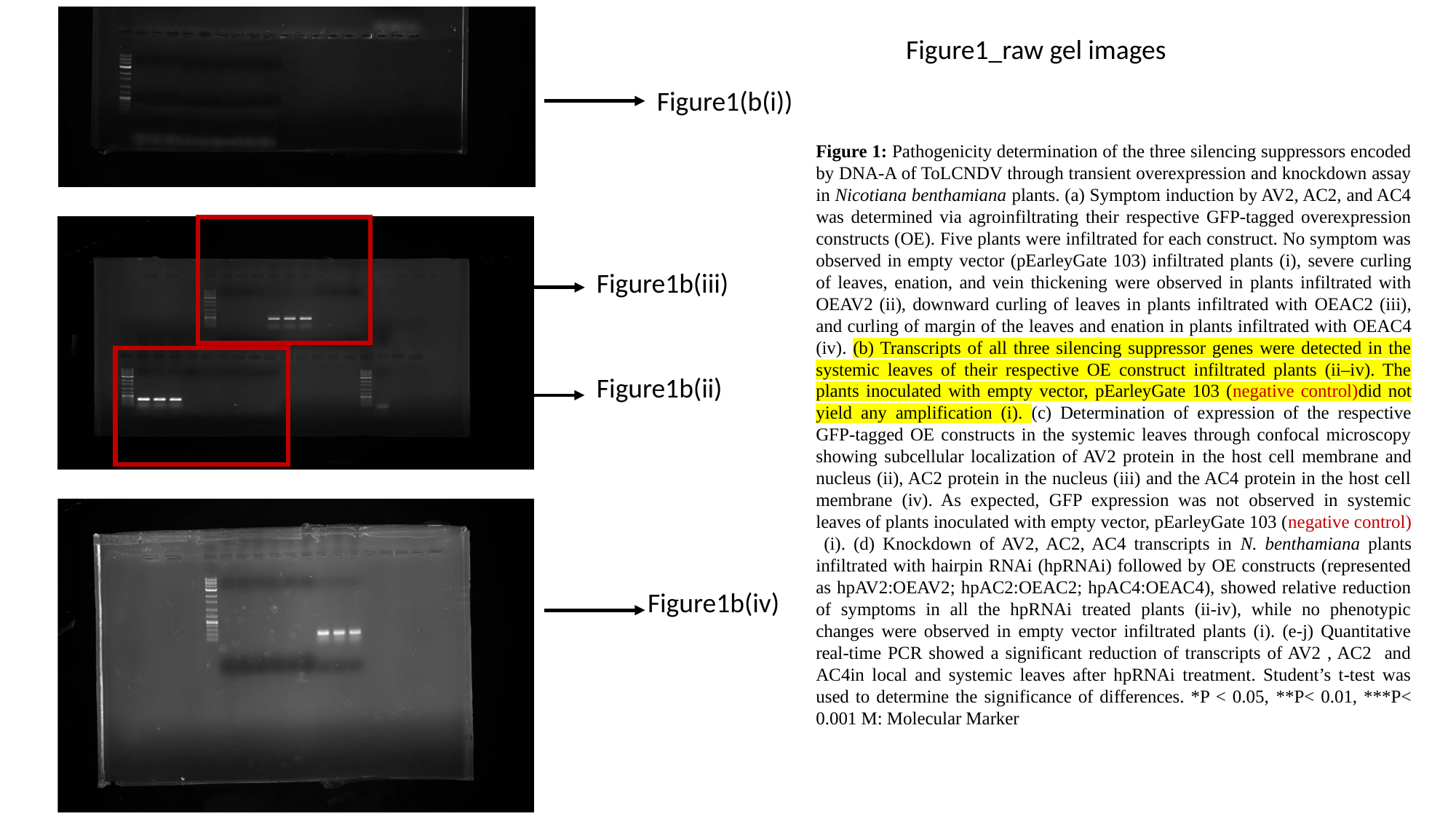

Figure1_raw gel images
Figure1(b(i))
Figure 1: Pathogenicity determination of the three silencing suppressors encoded by DNA-A of ToLCNDV through transient overexpression and knockdown assay in Nicotiana benthamiana plants. (a) Symptom induction by AV2, AC2, and AC4 was determined via agroinfiltrating their respective GFP-tagged overexpression constructs (OE). Five plants were infiltrated for each construct. No symptom was observed in empty vector (pEarleyGate 103) infiltrated plants (i), severe curling of leaves, enation, and vein thickening were observed in plants infiltrated with OEAV2 (ii), downward curling of leaves in plants infiltrated with OEAC2 (iii), and curling of margin of the leaves and enation in plants infiltrated with OEAC4 (iv). (b) Transcripts of all three silencing suppressor genes were detected in the systemic leaves of their respective OE construct infiltrated plants (ii–iv). The plants inoculated with empty vector, pEarleyGate 103 (negative control)did not yield any amplification (i). (c) Determination of expression of the respective GFP-tagged OE constructs in the systemic leaves through confocal microscopy showing subcellular localization of AV2 protein in the host cell membrane and nucleus (ii), AC2 protein in the nucleus (iii) and the AC4 protein in the host cell membrane (iv). As expected, GFP expression was not observed in systemic leaves of plants inoculated with empty vector, pEarleyGate 103 (negative control) (i). (d) Knockdown of AV2, AC2, AC4 transcripts in N. benthamiana plants infiltrated with hairpin RNAi (hpRNAi) followed by OE constructs (represented as hpAV2:OEAV2; hpAC2:OEAC2; hpAC4:OEAC4), showed relative reduction of symptoms in all the hpRNAi treated plants (ii-iv), while no phenotypic changes were observed in empty vector infiltrated plants (i). (e-j) Quantitative real-time PCR showed a significant reduction of transcripts of AV2 , AC2 and AC4in local and systemic leaves after hpRNAi treatment. Student’s t-test was used to determine the significance of differences. *P < 0.05, **P< 0.01, ***P< 0.001 M: Molecular Marker
Figure1b(iii)
Figure1b(ii)
Figure1b(iv)

## Slide 2
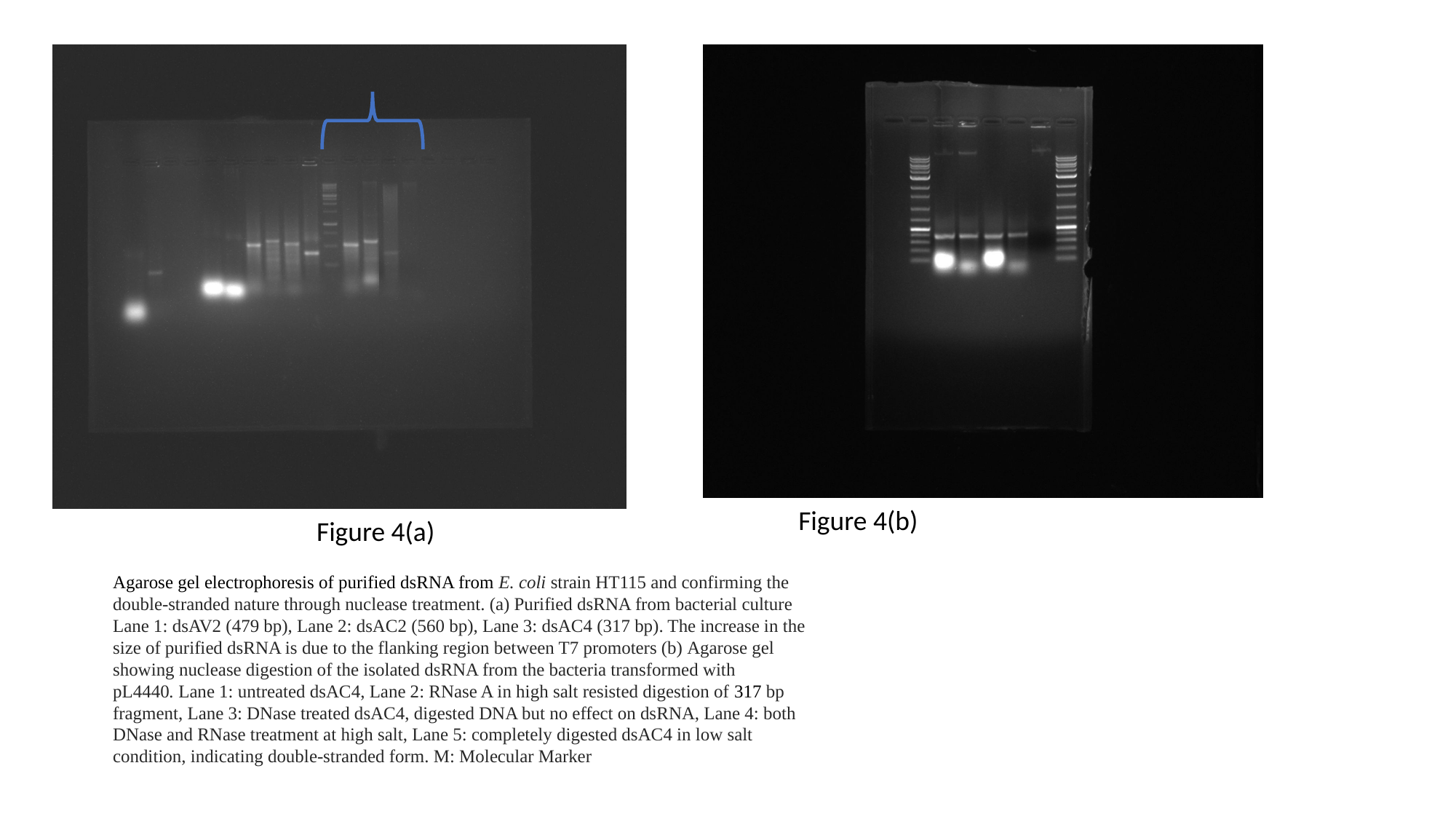

Figure 4(b)
Figure 4(a)
Agarose gel electrophoresis of purified dsRNA from E. coli strain HT115 and confirming the double-stranded nature through nuclease treatment. (a) Purified dsRNA from bacterial culture Lane 1: dsAV2 (479 bp), Lane 2: dsAC2 (560 bp), Lane 3: dsAC4 (317 bp). The increase in the size of purified dsRNA is due to the flanking region between T7 promoters (b) Agarose gel showing nuclease digestion of the isolated dsRNA from the bacteria transformed with pL4440. Lane 1: untreated dsAC4, Lane 2: RNase A in high salt resisted digestion of 317 bp fragment, Lane 3: DNase treated dsAC4, digested DNA but no effect on dsRNA, Lane 4: both DNase and RNase treatment at high salt, Lane 5: completely digested dsAC4 in low salt condition, indicating double-stranded form. M: Molecular Marker

## Slide 3
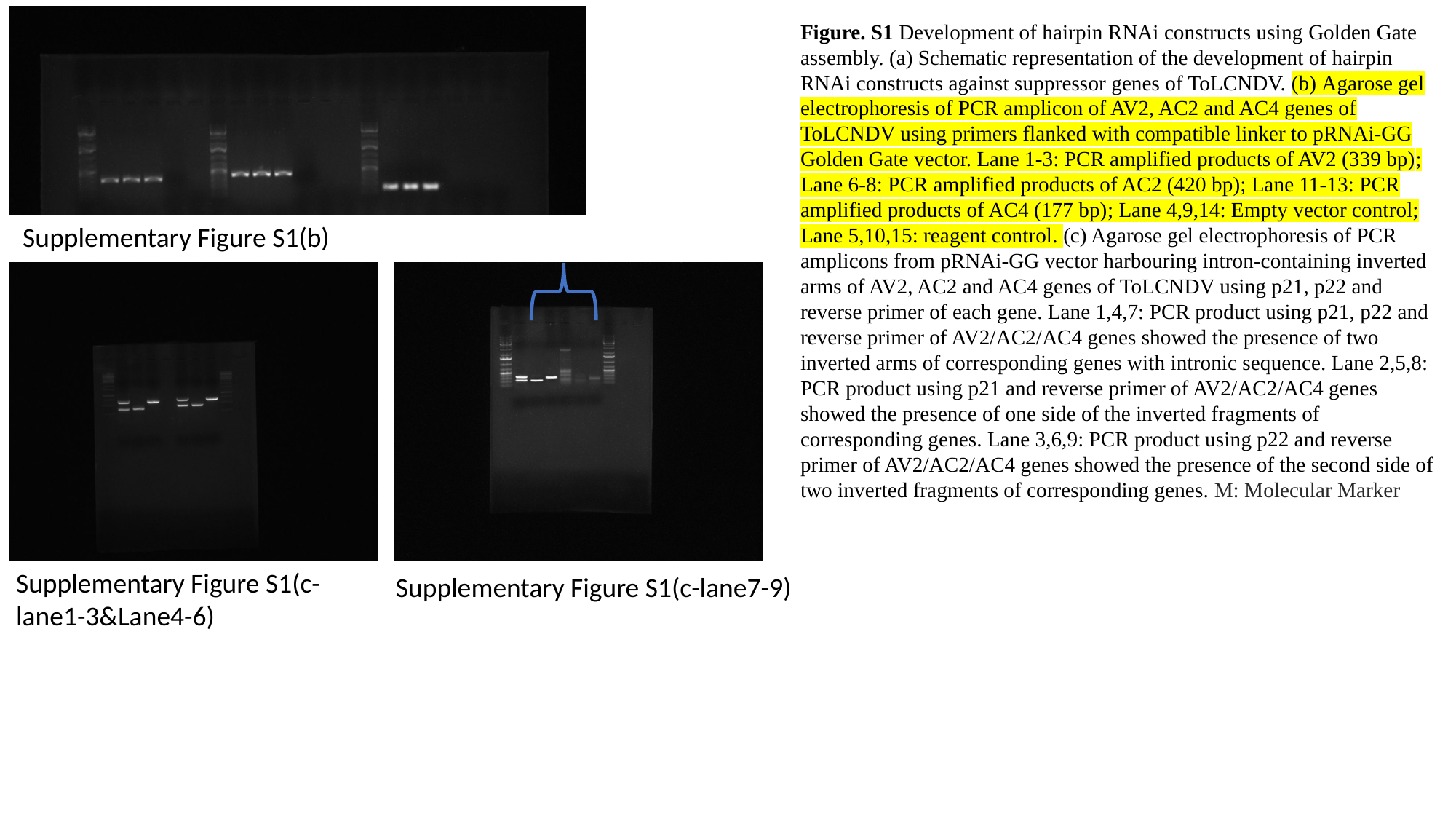

Figure. S1 Development of hairpin RNAi constructs using Golden Gate assembly. (a) Schematic representation of the development of hairpin RNAi constructs against suppressor genes of ToLCNDV. (b) Agarose gel electrophoresis of PCR amplicon of AV2, AC2 and AC4 genes of ToLCNDV using primers flanked with compatible linker to pRNAi-GG Golden Gate vector. Lane 1-3: PCR amplified products of AV2 (339 bp); Lane 6-8: PCR amplified products of AC2 (420 bp); Lane 11-13: PCR amplified products of AC4 (177 bp); Lane 4,9,14: Empty vector control; Lane 5,10,15: reagent control. (c) Agarose gel electrophoresis of PCR amplicons from pRNAi-GG vector harbouring intron-containing inverted arms of AV2, AC2 and AC4 genes of ToLCNDV using p21, p22 and reverse primer of each gene. Lane 1,4,7: PCR product using p21, p22 and reverse primer of AV2/AC2/AC4 genes showed the presence of two inverted arms of corresponding genes with intronic sequence. Lane 2,5,8: PCR product using p21 and reverse primer of AV2/AC2/AC4 genes showed the presence of one side of the inverted fragments of corresponding genes. Lane 3,6,9: PCR product using p22 and reverse primer of AV2/AC2/AC4 genes showed the presence of the second side of two inverted fragments of corresponding genes. M: Molecular Marker
Supplementary Figure S1(b)
Supplementary Figure S1(c-lane1-3&Lane4-6)
Supplementary Figure S1(c-lane7-9)
